# A Solitary Diffuse Neurofibroma in a Young Adult Female: An Unusual Presentation in the Scapular Region

**DOI:** 10.7759/cureus.90884

**Published:** 2025-08-24

**Authors:** Stephanie Saldaña Guerrero, Talissa F Garza Tovar, Quitzia L Torres

**Affiliations:** 1 Dermatology, IMSS Unidad Médica de Alta Especialidad (UMAE) No. 71, Torreón, MEX; 2 Dermatology, Universidad Juárez del Estado de Durango, Durango, MEX; 3 Biomedical Sciences, Universidad Juárez del Estado de Durango, Durango, MEX

**Keywords:** diffuse neurofibroma, histopathology, peripheral nerve sheath tumor, scapular region, solitary lesion

## Abstract

Diffuse neurofibroma is a benign peripheral nerve sheath tumor characterized by non-encapsulated spindle cell proliferation within the dermis and subcutaneous tissue. Although usually associated with neurofibromatosis type I (NF1) and preferentially located in the head and neck of pediatric patients, rare instances of sporadic solitary cases in atypical locations have been documented. We describe the case of a 30-year-old female with no personal or familial signs of neurocutaneous syndromes who presented with a two-year history of a slowly enlarging, asymptomatic plaque on the left scapular region. Dermatologic examination revealed an edematous, skin-colored plaque with multiple soft papillary projections. Histopathological analysis showed a poorly circumscribed proliferation of S-shaped spindle cells within a myxoid stroma, consistent with diffuse neurofibroma. Surgical excision was recommended due to infiltrative growth and margin involvement. The presentation of diffuse neurofibroma as a solitary scapular lesion in an adult without stigmata of NF1 is exceptional. Clinical morphology may mimic other benign dermal proliferations, highlighting the importance of histopathological confirmation. The infiltrative behavior and positive margins require complete surgical removal to minimize recurrence risk. Diffuse neurofibroma should be considered in the differential diagnosis of plaque-like lesions with soft nodularity, even in the absence of systemic features of NF1. Histopathologic evaluation is essential for an accurate diagnosis and adequate management planning.

## Introduction

Neurofibroma is a benign peripheral nerve sheath tumor composed of Schwann cells, fibroblasts, and perineurial-like cells. While localized and plexiform variants are more commonly described, the diffuse subtype represents a rare entity that typically affects the head and neck region in children or young adults and is often associated with neurofibromatosis type 1 (NF1) [1]. Unlike the localized type, diffuse neurofibromas tend to infiltrate the dermis and subcutaneous tissue in a plaque-like distribution, sometimes resembling soft fibromas or lipomatous tumors [2]. Although most cases of diffuse neurofibroma are linked to NF1, and less frequently to neurofibromatosis type 2 (NF2), sporadic cases constitute only about 10% of the total. Truly isolated cases not associated with either NF1 or NF2 are exceedingly rare, underscoring the unusual nature of our patient’s presentation [3].

Although diffuse neurofibromas are generally observed in the periorbital or scalp regions, their presentation as a solitary lesion in the scapular area of an otherwise healthy adult woman without NF1 stigmata is exceedingly rare. Histologically, these lesions are poorly circumscribed, unencapsulated, and composed of S-shaped spindle cells within a myxoid stroma, often requiring biopsy for a definitive diagnosis [4]. We present the case of a 30-year-old woman with a solitary edematous plaque on the left scapular region, histologically consistent with diffuse neurofibroma, highlighting the diagnostic challenges and the importance of histopathological evaluation in atypical presentations.

## Case presentation

**Figure 1:**
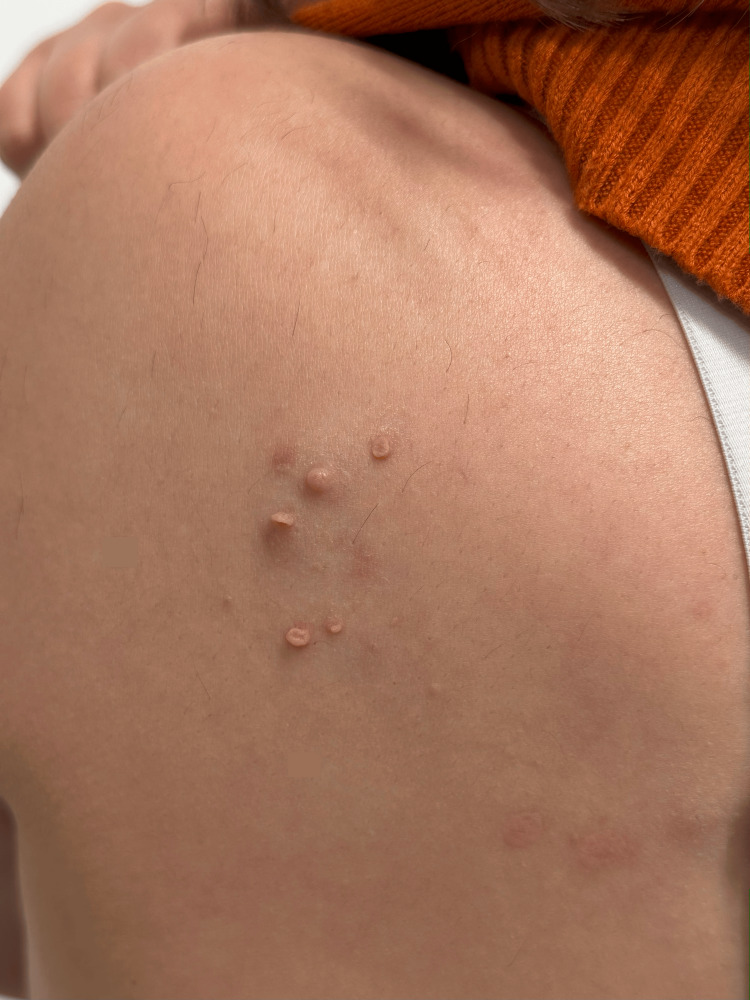
Edematous, skin-colored plaque with soft, pinkish projections on the left scapular region

A 30-year-old female with no relevant family history of neurocutaneous syndromes presented with a two-year history of a gradually enlarging, asymptomatic cutaneous lesion over the left scapular region (Figure 1). Her past medical history included Raynaud’s phenomenon and postpartum depression managed with duloxetine and alprazolam. She denied tobacco, alcohol, or recreational drug use.

Dermatological examination revealed a dermatosis localized to the left scapular region, consisting of an edematous plaque of skin-colored hue with poorly defined borders; on its surface, five soft, pinkish neoformations resembling soft fibromas were observed. No café-au-lait macules, axillary freckling, Lisch nodules, or other cutaneous stigmata of NF1 were observed.

A punch biopsy was performed. Histopathological analysis demonstrated basket-weave hyperkeratosis, an undulating epidermal architecture, and multifocal basal layer pigmentation. Within the superficial to mid-reticular dermis, there was a poorly circumscribed, non-encapsulated proliferation of spindle cells with pale eosinophilic cytoplasm and wavy, S-shaped nuclei arranged in short, disorganized fascicles. Scattered mast cells and congested capillaries were noted throughout the lesion. Foci of neural differentiation were also present. The lesion extended to the surgical margins.

**Figure 2:**
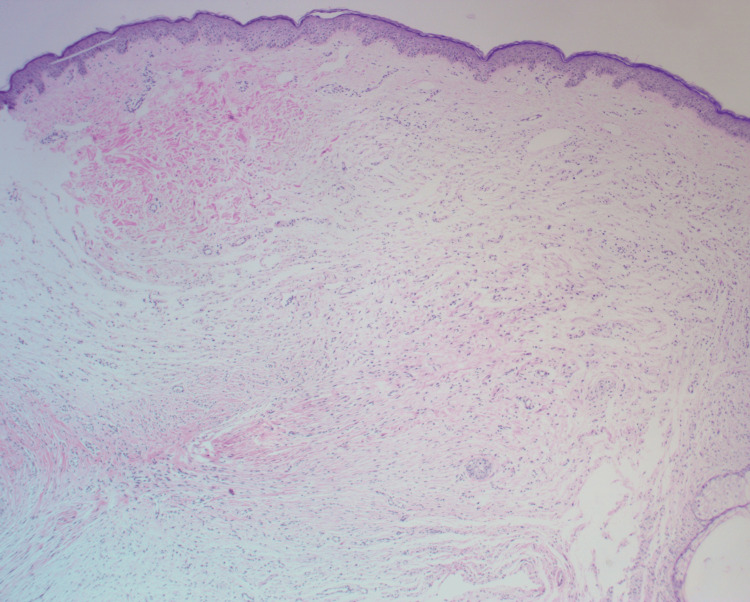
Low-power view (H&E, 4×) showing a proliferation of spindle-shaped cells within the mid to deep reticular dermis

**Figure 3:**
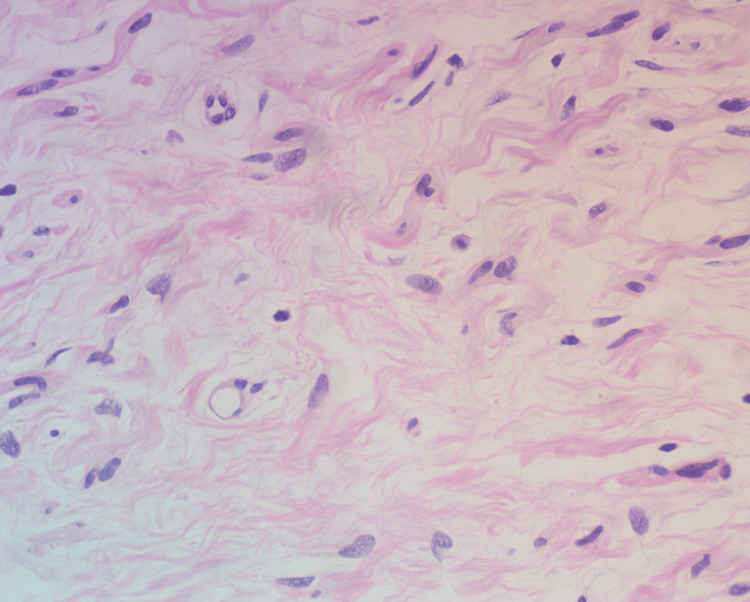
High-power view (H&E, 40×) demonstrating tumor extension in short, disorganized fascicles interspersed among thick collagen bundles and embedded in a myxoid stroma The proliferating cells exhibit spindle morphology with blunt-ended, tapered nuclei and abundant pale eosinophilic cytoplasm. Numerous mast cells are evident between the fascicles

Low-power examination (Figure 2) revealed a spindle-cell proliferation within the mid to deep reticular dermis, arranged in loose fascicles interspersed among thick collagen bundles and embedded in a myxoid stroma. At higher magnification (Figure 3), the proliferating cells exhibited blunt-ended, tapered nuclei and abundant pale eosinophilic cytoplasm. Numerous mast cells were evident, interspersed between the fascicles, supporting the diagnosis of a neurofibromatous lesion.

These findings were consistent with diffuse neurofibroma. No systemic signs suggestive of NF1 were observed during follow-up. The patient was referred to surgical oncology for complete excision due to the lesion’s histological infiltrative nature and margin involvement.

## Discussion

Diffuse neurofibroma is an uncommon benign peripheral nerve sheath tumor, most frequently associated with NF1, and typically seen in children and young adults. However, several case series and reports have documented sporadic presentations in adults lacking systemic features or a family history of NF1, underscoring the diagnostic importance of recognizing atypical clinical scenarios [5]. As described by Bower et al., the hallmark histological features of diffuse neurofibroma include a non-encapsulated proliferation of bland spindle cells with pale eosinophilic cytoplasm and S-shaped nuclei, arranged in short, disorganized fascicles within a myxoid stroma. Scattered mast cells and ectatic capillaries are frequently present, and areas of neural differentiation may be observed [6]. These features were fully consistent with the findings in our patient, whose lesion extended to the lateral and deep surgical margins, indicating the infiltrative nature typical of this tumor subtype.

Huang et al. have reported that diffuse neurofibromas often lack a well-defined border and may mimic lipomatous or vascular tumors, particularly when occurring in less common locations such as the trunk or limbs [5]. Although advanced imaging was not performed in our case, the clinical morphology (a mildly edematous plaque with soft, papillomatous projections) could have potentially led to a misdiagnosis in the absence of histological confirmation. Khatri et al., in their multi-case series, emphasized the diagnostic challenge posed by diffuse neurofibromas due to their variable clinical presentation, especially in the absence of NF1 stigmata. In their experience, definitive diagnosis often required biopsy, and many patients underwent debulking surgery for functional or cosmetic reasons, even when NF1 diagnostic criteria were not met [7]. This observation aligns with our case, where surgical excision was recommended following histologic diagnosis, given the infiltrative growth pattern and the frequent involvement of surgical margins that characterize this tumor subtype.

Further supporting this diagnostic complexity, Levy Bencheton et al. reported an unusual case of a solitary late-onset plexiform neurofibroma in an 85-year-old patient without personal or familial history of NF1 [8]. Similar to our patient, the lesion exhibited deep subcutaneous extension and an infiltrative growth pattern, which hindered complete surgical excision and led to repeated recurrences. Of note, their case showed a prominent myofibroblastic component (an uncommon histological feature not observed in our specimen) that may complicate histopathological interpretation, as it can mimic reactive fibrosis or scar tissue [8].

Additional diagnostic challenges are highlighted in the case described by Ebrahim et al., in which a diffuse neurofibroma arising in the parotid gland was initially misdiagnosed as a pleomorphic adenoma based on imaging and cytology [9]. The true diagnosis - low-grade malignant peripheral nerve sheath tumor (MPNST) arising within a diffuse neurofibroma - was only established after surgical resection and thorough histopathological evaluation. This case underscores the importance of obtaining tissue for definitive diagnosis, particularly in lesions with atypical features, and highlights the rare but significant potential for malignant transformation. Although our case lacked histological evidence of malignancy, the lesion’s size and infiltrative margins support the need for close clinical monitoring.

Similarly, Jung et al. documented a superficial MPNST arising from a recurrent neurofibroma in the abdominal wall of a patient without clinical features of NF1 [10]. This case illustrates that even sporadic neurofibromas, particularly those that recur, may carry a rare risk of malignant transformation. Reported rates of malignant change in neurofibromas overall remain low, estimated at less than 5%, and are most often described in association with NF1. However, isolated cases in non-syndromic patients have been published, underscoring the importance of clinical vigilance. Histologically, malignant transformation is usually characterized by features consistent with MPNST, including hypercellularity, nuclear atypia, increased mitotic activity, and necrosis. Although such progression is exceptional in the absence of syndromic NF1, these reports reinforce the need for long-term follow-up and heightened clinical suspicion in patients with recurrent or atypically growing lesions.

Taken together, these cases (including ours) contribute to the growing body of literature challenging the traditional notion of diffuse neurofibroma as a hallmark of NF1. Instead, they highlight the potential of these tumors to arise sporadically, display histological variability, mimic other soft tissue neoplasms, and, in some instances, evolve toward malignancy. These overlapping clinical and histopathological features may lead to considerable diagnostic difficulties, underscoring the necessity of biopsy and thorough pathological evaluation. Although considered benign, diffuse neurofibromas warrant careful pathological assessment and long-term clinical vigilance due to their potential for local invasion, recurrence, and, albeit rarely, malignant transformation.

## Conclusions

Diffuse neurofibroma should be considered in the differential diagnosis of slowly enlarging, plaque-like cutaneous lesions with soft nodularity, even in the absence of NF1 features. The lesion’s infiltrative histology and margin involvement underscore the necessity of complete surgical excision and close follow-up to reduce the risk of recurrence. Given the potential for clinical misidentification, histopathological confirmation remains essential for accurate diagnosis and appropriate management planning in these patients.
